# Intraosseous Hibernoma: A Rare Entity in Orthopedics With Peculiar Radiological Features

**DOI:** 10.7759/cureus.39883

**Published:** 2023-06-02

**Authors:** Ramy Samargandi, Louis-Romée Le Nail, Gonzague de Pinieux, Matthias Tallegas, Elodie Miquelestorena-Standley

**Affiliations:** 1 Service d’Orthopédie et Traumatologie, Centre Hospitalier Régional Universitaire (CHRU) de Tours, Tours, FRA; 2 Orthopedic Surgery Department, Faculty of Medicine, University of Jeddah, Jeddah, SAU; 3 Service d’Anatomie et Cytologie Pathologique, Centre Hospitalier Régional Universitaire (CHRU) de Tours, Tours, FRA; 4 Service Biologie Moléculaire, Centre Hospitalier Régional Universitaire (CHRU) de Tours, Tours, FRA

**Keywords:** benign tumors, bone tumors, brown fat cells, hibernoma, intraosseous hibernoma

## Abstract

Intraosseous hibernoma is a rare benign bone tumor derived from brown fat. It is typically found in the axial skeleton and is more commonly observed in women. It can manifest as a painful lesion or may be incidentally discovered. Intraosseous hibernoma often presents as a sclerotic lesion, although it can also manifest as a lytic lesion. Due to its varied radiographic appearance, it should be considered in the differential diagnosis of bone lesions as it can mimic metastatic lesions as well as other sclerotic and lytic bone lesions. Therefore, obtaining a biopsy of the lesion is crucial for an accurate diagnosis. In this report, we present the clinical, radiological, and histopathological findings of two cases of intraosseous hibernoma and provide a concise overview based on a review of the literature.

## Introduction

Hibernoma is an uncommon benign neoplasm composed of adipocytic cells containing brown fat, typically manifesting as a painless, indolent mass occurring in the soft tissues. The tumor primarily occurs in the thigh, followed by the trunk and upper limb, with no apparent gender predisposition. Its incidence peaks during the third and fourth decades of life [1]. Histopathologically, hibernoma exhibits distinctive features characterized by multivacuolated adipocytes displaying eccentrically positioned nuclei and lacking atypia. These cells closely resemble brown fat cells intermixed with multivacuolated adipocytes observed in lipomas. Notably, positive immunostaining for S100 protein can be observed in hibernoma [2].

Recently, intraosseous hibernoma (IOH) has been reported. Thorn initially reported this entity in 2008 [3]. Subsequently, several studies have documented cases of IOH at various anatomical locations, with the axial skeleton being the most frequently affected site. To the best of our knowledge and as of the present study, a total of 23 studies encompassing 37 cases have been described in the literature [3-25]. This current study contributes two additional cases of intraosseous hibernoma involving the manubrium sterni and iliac crest, expanding our understanding of its clinical presentation. Furthermore, a comprehensive evaluation of the clinical, radiological, and histological aspects of all previously reported cases is provided in this study, aiming to enhance the existing knowledge of this rare entity.

## Case presentation

Case 1

**Figure 1:**
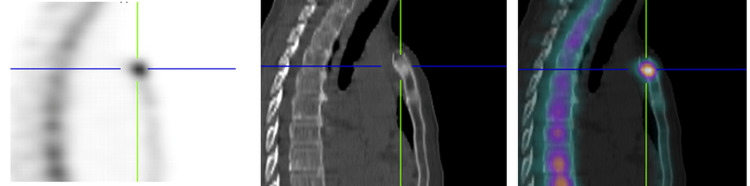
SPECT-CT imaging revealed an increased uptake within the lesion and a sclerotic appearance without any evidence of cortical destruction in the manubrium sterni. SPECT-CT: single-photon emission computed tomography

**Figure 2:**
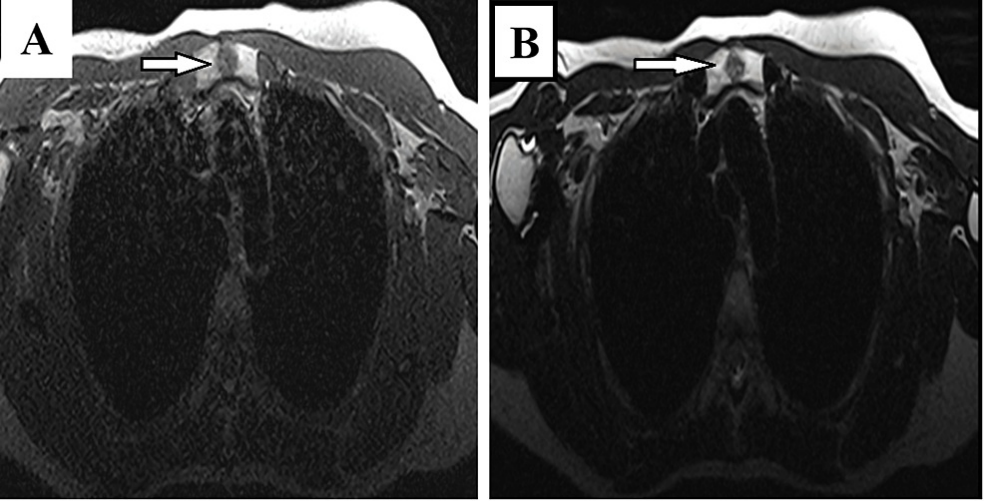
MRI imaging demonstrating the lesion in the manubrium sterni (white arrow). (A) Axial T1-weighted image reveals an intermediate signal intensity resembling muscles. (B) Axial T2-weighted image displays a heterogeneous lesion with mild hyperintensity.

**Figure 3:**
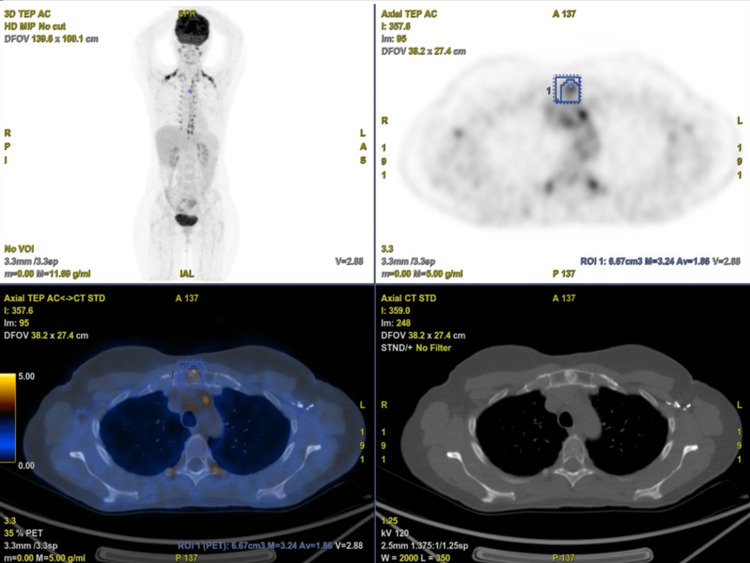
A FDG-PET scan demonstrates mild uptake with a maximum standardized uptake value (SUVmax) of 3.2. FDG-PET: fluorodeoxyglucose-positron emission tomography

A 49-year-old female patient with no significant medical history was recently diagnosed with bilateral breast cancer. During the staging process, an incidental sternal bony lesion was identified, raising concerns about possible bone metastasis. Radiological evaluation using single-photon emission computed tomography (SPECT) revealed a 13-mm sclerotic lesion in the sternal manubrium, exhibiting hyperfixation without evidence of cortical erosion or visible periosteal apposition (Figure 1). Magnetic resonance imaging (MRI) demonstrated low signal intensity in both T1- and T2-weighted sequences, with early contrast enhancement following contrast injection (Figure 2). Additionally, fluorodeoxyglucose positron emission tomography (FDG PET) exhibited increased tracer uptake in the lesion, with a standardized uptake value (SUV) of 3.2 (Figure 3). Given the possibility of a secondary lesion, a surgical biopsy was performed to determine the nature of the sternal lesion.

**Figure 4:**
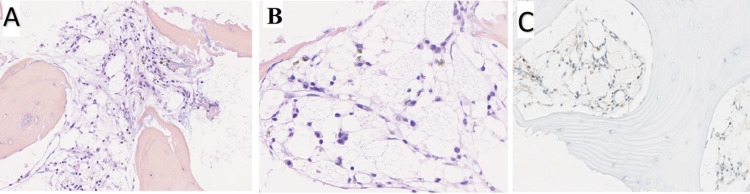
The histopathological appearance of the biopsy specimen confirms the diagnosis of intraosseous hibernoma. (A) At low magnification (x100), microscopic examination reveals a population of large cells with foamy cytoplasm, accompanied by dilated capillaries between trabeculae of thickened lamellar bone (hematoxylin and eosin staining). (B) At higher magnification (x400), the foamy cells exhibit a microvacuolar cytoplasm with centrally or para-centrally located nuclei. (C) Immunohistochemistry demonstrates positive expression of the S100 protein (x100 magnification).

Microscopic examination revealed a sclerotic trabecular bone structure, along with clear areas resembling medullary spaces. These clear areas consisted of large cells displaying cytoplasmic microvacuolations reminiscent of brown fat cells. Immunophenotyping analysis demonstrated the clear cells to be positive for the S100 protein (Figure 4), while they were negative for pankeratin AE1-1E3, CD163, and brachyury.

Case 2

**Figure 5:**
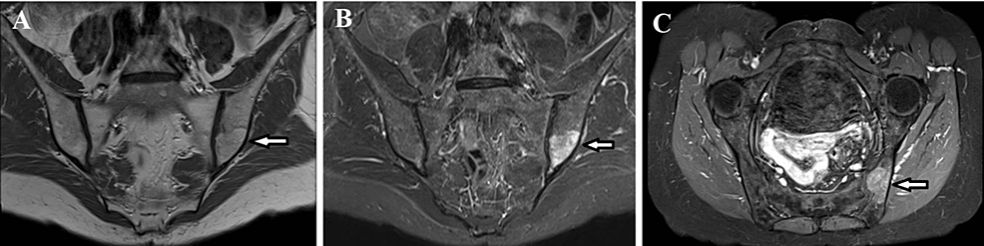
MRI imaging demonstrates the lesion in the left iliac bone (white arrows). (A) Coronal T1-weighted image shows a moderately high signal intensity, appearing brighter than the bone marrow and lower than subcutaneous fat. (B) Coronal short tau inversion recovery (STIR) sequence demonstrates a high-signal, ill-defined, and heterogeneous lesion. (C) Axial T1-weighted image with fat suppression reveals contrast enhancement after gadolinium injection.

**Figure 6:**
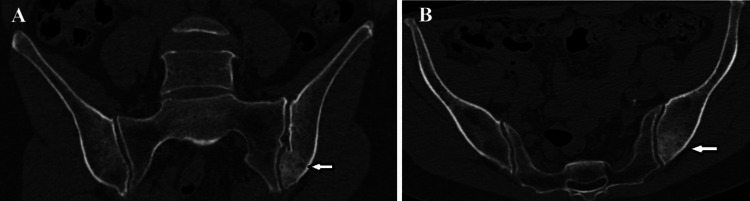
CT scan demonstrating the lesion in the left iliac bone (white arrows). (A) Coronal CT scan showing a sclerotic, ill-defined lesion within the left posterior ilium. (B) Axial CT scan showing a sclerotic, ill-defined lesion with no sclerotic rim in the left posterior ilium.

A 63-year-old woman with no prior medical history was referred by her family physician due to an incidental bony lesion identified on MRI during the evaluation of chronic back pain and left sciatica (Figure 5). The patient reported intermittent left sciatica with prolonged walking but had no neurological deficits. A CT scan revealed an ill-defined, mildly sclerotic lesion in the left iliac bone adjacent to the sacroiliac joint (Figure 6). Laboratory tests yielded normal results. A percutaneous CT-guided biopsy was performed to rule out infection or primary and secondary bone lesions.

**Figure 7:**
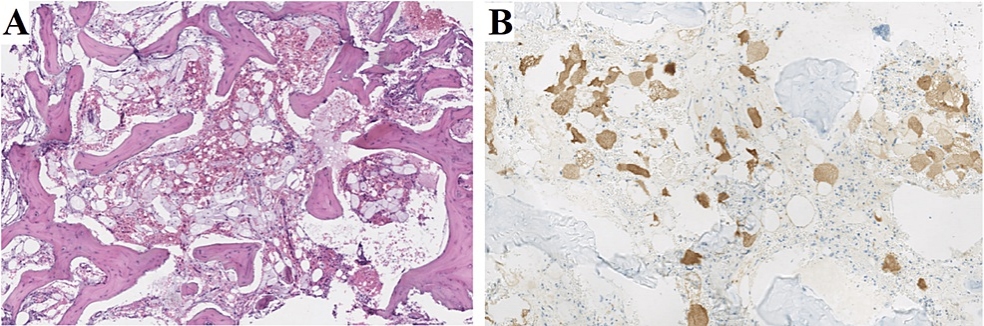
Histopathology demonstrates intraosseous hibernoma. (A) At high magnification, histologic examination revealed the presence of large cells with microvacuolated cytoplasm in the spaces between the bone trabeculae, resembling brown fat cells. The cytoplasmic appearance was accompanied by capillaries and leukocytes (hematoxylin and eosin staining, magnification x400). (B) The immunohistochemical study showed cytoplasmic and nuclear staining of the clear cell population, consistent with adipose tissue. This staining was observed using S100 protein as the primary antibody (DAKO polyclonal antibody), further supporting the diagnosis (Magnification x100).

The histological examination of the biopsy specimen demonstrated similar characteristics to the first case, including abnormal bone tissue composed of thick interconnected trabeculae separated by clear spaces containing numerous capillaries and clear cells with multivacuolated pale cytoplasm and a central or paracentral nucleus. Immunohistochemical analysis revealed positive expression of the S100 protein in the clear cells, while epithelial, histiocytic, and brachyury markers showed negative staining (Figure 7).

## Discussion

Brown fat cells are characterized by their high vascularity and abundance of mitochondria, giving them the appearance of a brown color. Typically found in newborns, they gradually diminish with age and transform into white fat cells. However, remnants of brown fat tissue can persist in adults, predominantly in the upper trunk and paravertebral regions. Brown fat plays a significant role in thermoregulation in newborns and has emerged as a potential target for addressing obesity and diabetes in adults [1].

Hibernoma is a benign tumor composed of brown adipocytes that primarily occurs in soft tissues. Intraosseous hibernoma (IOH) is extremely rare and has only recently been described. The first report of IOH was documented by Thorn et al. in 2008, and subsequent cases have been reported in the literature [3-25].

To the best of our knowledge, including the two cases presented in this study, a total of 39 cases of IOH have been reported. IOH is typically observed in adults between the ages of 36 and 85, with an average age of 59.4 years. It occurs more frequently in women (69.2%) than in men (30.8%), with a female-to-male ratio of 2.1:1. In contrast, soft tissue hibernomas are more commonly found in men [1]. Consistent with this pattern, the two cases reported in our study involved female patients. IOH also tends to manifest in older populations compared to soft tissue hibernomas, with an average age difference of 21.2 years (59.2 years for IOH versus 38.0 years for soft tissue hibernomas) [2].

IOH is most commonly found in the axial skeleton and pelvis, with the sacrum being the most common site (33.3%), followed by the hip bone (27.0%) and vertebral bodies (23.1%). Other locations, such as the femur, ribs, and sternum, are less frequently involved. The majority of IOH cases (66.6%) are incidentally discovered, often during bone marrow aspiration for hematologic evaluations or through imaging investigations for cancer staging or unrelated back pain. However, 14 patients (37.8%) among the available clinical data presented with pain (Table 1).

**Table 1:** Demographic, clinical, and radiological presentation of IOH regarding the site of the lesion among 39 reported cases, including the present study. *Among two available clinical data and one available radiological data # Among the seven available radiological data

Location	Number of cases (%)	Number of osteolytic lesions among all lesions (%)	Number of Sclerotic lesions among all lesions (%)	Number of occult lesions among all lesions (%)	Number of painful lesions (%)	Number of incidental finding (%)
Total number		6 (17.6)	26 (76.4)	2 (5.8)	14 (37.8)	22 (66.6)
Sacrum	13 (29.7)	5 (38.4)	4 (46.1)	2 (15.3)	6 (46.1)	7 (53.8)
Hip bone	10 (27)	0	7 #	0	2 (20)	8 (80)
Vertebra	9 (24.3)	0	9 (100)	0	3 (33.3)	6 (66.6)
Manubrium sterni	3 (8.1)	0	3 (100)	0	0	3 (100)
Femur *	3 (8.1)	0	1	0	1	1
Ribs	1 (2.7)	1 (100)	0	0	1 (100)	0
Total number of available data among 39 reported cases, including the present study		34	34	34	37	37

There may be a correlation between the radiological and clinical features based on the location of the lesion. Among the reported osteolytic lesions, one occurred in the fourth rib, while the remaining five were in the sacrum (38.4% of sacral lesions presenting as osteolytic). On the other hand, all vertebral, hip, and sternal lesions were sclerotic. These findings suggest that sacral lesions may exhibit distinct radiological presentations compared to other common sites. However, due to the rarity of this condition and the limited published data, this hypothesis cannot be confirmed. Table 1 provides an overview of the anatomical distribution in relation to symptoms and radiological features.

There are no pathognomonic radiological features specific to intraosseous hibernoma (IOH). Therefore, a biopsy of the lesion is crucial for making an accurate diagnosis. Among the cases with available radiological data (34 cases), IOH most commonly appears as a nonspecific sclerotic lesion (in 26 cases, 76.4%) on CT scans, mimicking conditions such as osteomyelitis, metastasis, lymphoma, and other benign sclerotic bone lesions like benign non-ossifying fibroma, bone island, hemangioma, and fibrous dysplasia. However, IOH can also present as an occult lesion (in two cases, 5.8%) or an osteolytic lesion (in six cases, 17.6%), with or without thin sclerotic margins. Cortical erosion or periosteal reaction was not observed in any of the reported cases.

On MRI, IOH exhibits T1 hypointensity relative to subcutaneous fat and hyperintensity relative to muscles, heterogeneous hyperintensity on T2-weighted images, and variable enhancement on post-contrast fat-saturated T1-weighted images. Additionally, IOH may demonstrate minimal to prominent uptake on bone scans [7,9,18,22] and mildly increased uptake on 18F-FDG PET scans with SUV ranging from 2.5 to 4.6 [9,11,15,21,24,25]. These imaging findings can be attributed to the increased vascularity, abundant mitochondria, and high rate of glucose metabolism in brown fat cells. Notably, there is a case report where 68Ga-DOTATATE PET was utilized during the staging of neuroendocrine tumors and incidentally detected an intense uptake (SUV 12.4 with 68Ga-DOTATATE versus SUV 4.6 with 18F-FDG) in an intraosseous hibernoma [21].

In our cases, the radiological evaluations were consistent with previously reported cases. Both cases exhibited sclerotic lesions on CT scans and T1 hypointense and T2 heterogeneous hyperintense lesions on MRI. Additionally, only one patient underwent FDG-PET, which was performed during breast cancer staging, revealing minimal uptake (SUV 3.2) in the IOH lesion.

Histologically, intraosseous hibernoma (IOH) is characterized by a varying number of eosinophilic to clear vacuolated granular cells called physaliferous-like cells, which have central or paracentral nuclei resembling brown fat cells. These cells can occasionally be mixed with uni-vacuolated adipocytes. However, it is important to consider interosseous lipoma as a potential differential diagnosis during histopathological examination. Immunohistochemical studies are useful in ruling out other differential diagnoses, including metastasis from renal cell carcinoma (positive for epithelial markers like cytokeratins AE1/AE3), histiocytosis (positive for histiocytic markers such as CD68 and CD163), and chordoma (positive for brachyury). Our patients exhibited similar histological findings to those previously reported.

The majority of IOH lesions do not require treatment, as they are often found incidentally. Among the available data for symptomatic reported cases (14 cases), most patients showed improvement in pain with medical management [5,7,16,18]. Five patients underwent interventional treatments, including one patient who underwent radiofrequency ablation (RFA) of the lesion with pain improvement [8], another patient who underwent microwave ablation (MWA) and cementoplasty [12,23], and three patients who underwent surgical removal of the lesion. However, these three surgical cases were performed without prior biopsy [10,15,17]. These findings suggest that IOH can be successfully managed through medical or surgical interventions.

Additionally, there is a reported case where RFA and kyphoplasty were performed simultaneously with a biopsy for an incidental lesion during ovarian cancer staging, which initially mimicked metastasis. Interventional procedures may be considered in cases of persistent disabling pain, regardless of medical management. Currently, there is limited data available to determine the optimal interventional treatment. However, percutaneous ablation appears to offer good pain improvement with less aggressiveness compared to surgical treatment. More reported cases in the future may help clarify the most effective treatment approach.

The question of whether intraosseous hibernoma is a neoplastic or physiological phenomenon remains unclear. Some authors suggest that these lesions could represent retained brown fat in adults rather than neoplastic lesions [3,6,9]. However, other authors believe that IOH is a benign neoplasm [5,7]. Based on the presence of radiologically distinct lesions and the clinical presentation of symptomatic cases in 37.8% of reported cases, which demonstrated improvement with medical or interventional treatment, we suggest that IOH is more likely a benign neoplasm than a physiological phenomenon. Further studies, including cytogenetic investigations, may help shed light on the exact etiology. It is worth noting that soft tissue hibernomas are associated with characteristic chromosomal rearrangements involving 11q13, but to our knowledge, no previous reports have conducted cytogenetic studies specifically for IOH [26].

## Conclusions

Intraosseous hibernoma is a rare benign lesion that has been recently described and should be considered as a potential differential diagnosis for sclerotic lesions, particularly in the axial skeleton. It is crucial to perform a biopsy to establish a definitive diagnosis and avoid unnecessary treatment, especially in cases where the lesion is discovered incidentally. This report contributes two additional cases that exhibit findings consistent with previously reported cases.

## References

[1] (2020). Soft Tissue and Bone Tumours Lyon.

[2] Furlong MA, Fanburg-Smith JC, Miettinen M (2001). The morphologic spectrum of hibernoma: a clinicopathologic study of 170 cases. Am J Surg Pathol.

[3] Thorns C, Schardt C, Katenkamp D, Kähler C, Merz H, Feller AC (2008). Hibernoma-like brown fat in the bone marrow: report of a unique case. Virchows Arch.

[4] Reyes AR, Wilson JD, Desai HS (2008). Intraosseous hibernoma of the femur: an unusual case with a review of the literature (poster #20). Coll Am Pathologists.

[5] Kumar R, Deaver MT, Czerniak BA, Madewell JE (2011). Intraosseous hibernoma. Skeletal Radiol.

[6] Lynch DT, Dabney RS, Andrews JM (2013). Intraosseous hibernoma or unusual location of brown fat?. J Hematop.

[7] Botchu R, Puls F, Hock YL (2013). Intraosseous hibernoma: a case report and review of the literature. Skeletal Radiol.

[8] Ringe KI, Rosenthal H, Länger F, Callies T, Wacker F, Raatschen HJ (2013). Radiofrequency ablation of a rare case of an intraosseous hibernoma causing therapy-refractory pain. J Vasc Interv Radiol.

[9] Bonar SF, Watson G, Gragnaniello C, Seex K, Magnussen J, Earwaker J (2014). Intraosseous hibernoma: characterization of five cases and literature review. Skeletal Radiol.

[10] Yahia M, Laabidi B, M’sakni I, Bougrine F, Bouziani A (2016). Intraosseous hibernoma: a case report and review of the literature. Tunis Med.

[11] Jerman A, Snoj Ž, Kuzmanov BG, Limpel Novak AK (2015). Intraosseous hibernoma: case report and tumor characterization. BJR Case Rep.

[12] Degnan AJ, Maldjian C, Pantanowitz L, Kofler JK (2015). Rare case of a radiographically occult sacral lesion detected on MRI presenting with intractable back pain. BJR Case Rep.

[13] Hafeez I, Shankman S, Michnovicz J, Vigorita VJ (2015). Intraosseous hibernoma: a case report and review of the literature. Spine (Phila Pa 1976).

[14] Dannheim K, Bhargava P (2016). A rare finding of brown fat in bone marrow as a mimic for metastatic disease. Am J Hematol.

[15] Vlychou M, Teh J, Whitwell D, Athanasou NA (2016). Intraosseous hibernoma: a rare adipocytic bone tumour. Skeletal Radiol.

[16] Westacott L, Collins A, Dickenson I (2016). Intraosseous hibernoma in the sacrum of an adult. Int J Surg Pathol.

[17] Georgiopoulos M (2016). Hibernoma: A Rare Intraosseous Tumor in the Illium.

[18] Song B, Ryu HJ, Lee C, Moon KC (2017). Intraosseous hibernoma: a rare and unique intraosseous lesion. J Pathol Transl Med.

[19] Chapman J, Vega F (2017). Incidental brown adipose tissue in bone marrow biopsy. Blood.

[20] Zuidberg-van der Gronde K, Gronde KZ van der, Klazen C (2017). A rare sternal lesion on magnetic resonance mammography mimicking a metastasis in a patient with a history of mamma carcinoma: a case report. J Med Cases.

[21] Woodford H, Le K, Bui C, Mansberg R (2019). Hibernoma demonstrated on 68Ga-DOTATATE PET/CT. Clin Nucl Med.

[22] Myslicki FA, Rosenberg AE, Chaitowitz I, Subhawong TK (2019). Intraosseous hibernoma: five cases and a review of the literature. J Comput Assist Tomogr.

[23] Ko A, Rowell CC, Vogler JB 4th, Samoilov DE (2020). Intraosseous hibernoma: a metastatic mimicker to consider on the differential. Radiol Case Rep.

[24] Bai S, Mies C, Stephenson J, Zhang PJ (2013). Intraosseous hibernoma: a potential mimic of metastatic carcinoma. Ann Diagn Pathol.

[25] Weiss SN, Mohla A, Zhu GG, Gutowski C, Kim TW, Amin R (2022). Intraosseous hibernoma: two case reports and a review of the literature. Radiol Case Rep.

[26] Maire G, Forus A, Foa C (2003). 11q13 alterations in two cases of hibernoma: large heterozygous deletions and rearrangement breakpoints near GARP in 11q13.5. Genes Chromosomes Cancer.

